# Commencements

**Published:** 1896-07

**Authors:** 


### COMMENCEMENTS.

### Ohio Medical University—Dental Department.

The fourth annual commencement exercises of the Ohio Medical University, including the Dental Department, were held in the Board of Trade Auditorium, Columbus, Ohio, on Tuesday evening, March 17, 1896.

The annual address was delivered by John B. Hamilton, M. D., LL.D., and the faculty address by George M. Waters, A. M., M. D.

The number of dental matriculates for the session was sixty-two.

The degree of D.D.S. was conferred on the following graduates by John M. Dunham, A.M., M.D., President of the Board of Trustees:

| NAME. | RESIDENCE. | NAME. | RESIDENCE. |
|---|---|---|---|
| Harry E Brubaker | Ohio | George L Miller | Pennsylvania |
| Augustus W Callinan | Ohio | Thomas M Patterson | Ohio |
| Harry Cope | Ohio | James F Richeson | Ohio |
| Abbot G Dana | Massachusetts | John J Stukey | Ohio |
| Elmer E Edenburn | Ohio | Owen H Thorpe | Ohio |
| Edwin S Fuller | Ohio | Ezra S Wagner | Ohio |
| George T Howard | Ohio | | |

### Chicago College of Dental Surgery.

The fourteenth annual commencement exercises of the Chicago College of Dental Surgery (Dental Department of Lake Forest University) were held in the Schiller Theater, Chicago, Ill., on Tuesday, April 7, 1896, at 2 P. M.

The doctorate address was delivered by C. N. Johnson, L.D.S., D.D.S., and the valedictory by William H. G. Logan, D.D.S., of the graduating class.

The number of matriculates for the session was four hundred and forty.

The degree of D.D.S. was conferred on the following graduates by Truman W. Brophy, M.D., D.D.S., LL.D., President of the College:

| NAME. | RESIDENCE. |
|---|---|
| John E Aigley | Illinois |
| Edward S Allen | Wisconsin |
| George Appelt | Germany |
| Oro DeG Babcock | Michigan |
| Augustus B Bailey | Oregon |
| Francis A Ballard | Iowa |
| Charles A Banghart | Canada |
| Samuel G Barker | Wisconsin |
| George T Boon | Kansas |
| Fred C Bradner | Indiana |
| Samuel E Burke | Michigan |
| Wm G Burkhardt | Illinois |
| George H Bush | Wisconsin |
| Albert O Boehmer | Canada |
| Reuben C Brophy, M.D | Illinois |
| William T Bell | Canada |
| William F Bevan | Illinois |
| Aksel T Bovesen | Minnesota |
| Edward F Caldwell | Illinois |
| Louis P Cardwell | Illinois |
| Russell V Cleveland | Illinois |
| John H Conant | Wisconsin |
| J H Cunningham | Wisconsin |
| F A Crookshank | Illinois |
| Herbert J Calkins | Wisconsin |
| Charles T Chandler | Wisconsin |
| John T Carpenter | Wisconsin |
| David St I Davies | North Dakota |
| Charles C Devereaux | Iowa |
| John B Dicus, B.S, A.B | Illinois |
| James Dodd, B.S | Illinois |
| William P Deurre | Minnesota |
| William H Dunn | Illinois |
| Charles C Dutton | Iowa |
| Albert E Eberhart | Minnesota |
| Timothy A Egan | Michigan |
| Jerome W Egbert | Illinois |
| George Eggers | Wisconsin |
| Joseph Eggers | Wisconsin |
| Charles J Fahsel | Illinois |
| John M Falvey | Wisconsin |
| Owen L Frazee | Illinois |
| John F Fribley | Illinois |
| Clyde C Fergusson | Canada |
| Edward H Goodsell | Minnesota |
| Matthew L Gregerson | Wisconsin |
| Wm S Griffiths, jr | Wisconsin |
| Augustus D Groshon | Illinois |
| D J F Hager | Canada |
| Arthur B Howatt | California |
| Frederick M Heiden | Wisconsin |
| Ashley M Hewett | Illinois |
| Fred J Holt | Colorado |
| Robert J Hood | Illinois |
| Charles H Hurlbut | Illinois |
| George E Huwatchek | Wisconsin |
| George Hulla | Illinois |
| George W Johnson | Illinois |
| George V Kohn | Wisconsin |
| Othello L Kerr | Missouri |
| Carl Klein, jr | Illinois |
| Floyd C Lander | Illinois |
| C O Letourneau | Illinois |
| John A Locheed | Illinois |
| W H G Logan | Illinois |
| Frank S Lombard | Illinois |
| Roderic S Maloney | Indiana |
| Benjamin F Martin | Illinois |
| Archibald McArthur | Wisconsin |
| Hugh W McMillan | Illinois |
| James D McMillan | Illinois |
| George H Madill | Canada |
| Charles S Methven | Illinois |
| Cornelius J Murphy | Illinois |
| Edward J Murray | Illinois |
| Neil P Nelson | Minnesota |
| Ingvald Nesheim | Norway |
| Sheldon Peck | Illinois |
| George W Pitts | Illinois |
| L G A Powell | Indiana |
| Michael J Prendergast | Canada |
| William A Quinn | Michigan |
| Wilbert C Reid | Michigan |
| Frederick W Rose | Canada |
| Alvah I Sargent | Illinois |
| Howard L Simmons | Illinois |
| Frank L Smith | Illinois |
| Claud H Snashall | Wisconsin |
| Frank F Snedecor | Alabama |
| LeRoy Snowden | Illinois |
| James H Steele | Kansas |
| Frederick W Stephan | Ohio |
| Albert B Stiles | Illinois |
| John A Stoeckley | Indiana |
| Alvin G Sturtz | Illinois |
| Wm L Selsor | Illinois |
| Gilbert R Tait | Wisconsin |
| James R Talpley | California |
| Mark W Trude | Illinois |
| Edward H Varnum | Illinois |
| John R Watt | Illinois |
| Frank A Weld | Illinois |
| Marshall G Wheeler | Alabama |
| Nelson B Winter | Iowa |
| Charles L Wyeth | Ohio |

### American College of Dental Surgery.

The tenth annual commencement exercises of the American College of Dental Surgery were held at the Grand Opera House, Chicago, Ill., on Monday, April 6, 1896, at 2:30 P. M.

The number of matriculates for the session was four hundred and twenty.

The doctorate address was delivered by Rev. P. S. Henson, D. D., the valedictory by Thomas G. Thompson, D.D.S., and the salutatory by Charles C. Lind, D.D.S.

The degree of D.D.S. was conferred on the following graduates by Henry Wade Rogers, D.D.S , President of the University:

Will O Asslen
William G Andrews
William Miller Ash
Walter Runnells Adams
Fred L Axtell
J F Boynton
J A Birchard
Carl S Byrnes
Joseph M Bischoff
A T Baxter
George M Berry
F H Bigness
H A Bear
John P Brunton
Charles Ellis Bartholf
F H Blaschka
James W Birkland
A G Bauer
Ellis R Boston
Polk Huntingdon Brown
A B Clark
J Truman Clark
William M Choate
Marcus H Cox
W E Carr
George M Dott
A John Davis
James E Dale
Marie Erickson
Francis J Freeman
W O Fellman
G H Frey
W J Ferguson
Arthur Paul Fillastre
Harry J Feltus
James R Goodrich
Samuel L Gants
Peter Gibson
D C S Garver
J C Gardiner
L Harrison Grove
W B Graham
Ray N Gibbs
L O Green
Sara Conklin Gramm
Mary Maloy Hawley
Frederick W Heineman
George S Hilliard
W B Hall
E V Harvey
Jacob C Hay
I B Howell
A W Head
F M Hole
John C Hamill
A J Hullinger
Charles H Jordan
L E James
J J Jackson
Samuel Jessup
D J Kuns
Bert H Kershaw
C L Kinney
Anna Marie Kirkeberg
Howard Hersey Kellogg
Herman Kuchler
C R Leidig
C Lincoln Lind
Duchamp Charles Labbe
Charles J Lyons
G D Libby
H E Macdonald
J Laurence Morris
H H Maynard
Arthur J Mears
Ida L Menges
J K Means
John McDermid
Alfred D McCabe
F E McCarthy
J N McDowell, B.S.
Murrray Lee Ong
M L Opheim, B.S.
D W Pratt
Samuel Hale Potter
A A Peterson, Ph.G.
A D Ratcliff
Francis G Richardson
G H Rolling
J H Reid
Anson F Poley
Frank Alexander Ross
Earle H Reaugh
J W Smith
Louis J Smith
O E Simpson
P D Silvernail
Charles E Stutenroth
Albert Stroebel
H H Straith
Wilber E Sackett
Edgar C Severns
M V Secrist
H C Spengler
T G Thompson
S A Turpin
L L Whitson
George E Wasser
S V Weiser
Clem White
Clark M Wilson
Louis M Williams
Harry Alexander Ware
L D Woltzen
G O Whitwam
Arthur E Young
Edmund L Yard
A O Yearian
A E Younkin
F B Young
J E Zipf
Emanuel Z Zipperman

### Western Dental College of Kansas City.

The sixth annual commencement exercises of the Western Dental College, of Kansas City, were held at the Auditorium, Kansas City, Mo., on Thursday evening, April 2, 1896.

The annual address was delivered by W. P. George, D.D.,

LL.D., the faculty address by W. F. Kuhn, A.M., M.D., and the valedictory by Thomas W. Lyell, D.D.S.

The number of matriculates for the session was two hundred and twenty.

The degree of D.D.S. was conferred on the following graduates by D. J. McMillen, M.D., D.D.S., Dean of the Faculty:

| NAME. | RESIDENCE. | NAME. | RESIDENCE. |
|---|---|---|---|
| Herman F Branstetter | Missouri | William Dillingham Kirby | Kansas |
| William Broadbent | Missouri | Frank Gilkeson Lobban | Missouri |
| Edwin Sever Brown | Missouri | Thomas Walter Lyell | Missouri |
| Edgar Pearson Brown | Missouri | Francis Marion McDonald | Texas |
| Clarence Manfred Burris | Iowa | Thomas McMillan | Missouri |
| Willis Victor Chapin | Kansas | Welby Stanley Morrow | Iowa |
| Christopher C Clark | Missouri | Joseph Shelby Mirick | Missouri |
| John Logan Clark | Missouri | Ralph Hammond McCrum | Missouri |
| Russell Elmer Covey | Missouri | Omar Preston Muckley | Ohio |
| Edward Abner Dabbs | Missouri | Orin Knisley Muckley | Ohio |
| George Whitman Downing | Missouri | John A Parker | Missouri |
| Fannie Delaney | Missouri | John Robert Pepper | Missouri |
| Albert Ulysses Edwards | Indiana | Henry Bosworth Purl | Missouri |
| Martin Frederick Ehlers | Missouri | Harley John Riley | Missouri |
| William Walter Flora | Kansas | Noah Richard Smith | Missouri |
| Thedore Green | Illinois | Theophilus Paul Smith | Missouri |
| Samuel Wilson Harris | Missouri | John August Steinmeyer | Kansas |
| Morton David Hamisfar | Missouri | James Campbell Thompson | Missouri |
| John Frank Huntling | Nebraska | John Elias Watkins | Kansas |
| Evan D Jenkins | Kansas | Charles Delosso Weakley | Missouri |
| George Francis Kelly | Kansas | Fred Emory Webster | Kansas |
| Richard Hiram Kent | Kansas | Linsey Leonidas Wills | Missouri |

### Royal College of Dental Surgeons of Ontario.

On the 21st of April the Directors of the Royal College of Dental Surgeons had under consideration the report of the Examiners for the session just closed, and have handed out the results as follows:

The number of students registered and in attendance for the session of 1895–96 was one hundred and sixty-one.

The following passed the final examinations and received the title of L.D.S. (Licentiate of Dental Surgery):

| | | |
|---|---|---|
| W F Adams | G W Hoag | C E Pearson |
| R M Armstrong | O H Hutchison | Percy Smith |
| T E Ball | J E Johnston | A T Sihler |
| F Britton | W E Lundy | J A Simpson |
| J J Brown | J L Leitch | W J Switzer |
| W Burnet | L M Mabee | J G Somerville |
| J M Bell | F S Mercer | W F Templar |
| L G Campbell | J F McMillan | W C Trotter, B.A. |
| H A Croll | H McQueen | Ed D Washington. |
| R N Henderson | | |

### Ohio College of Dental Surgery.

The fiftieth annual commencement exercises of the Ohio College of Dental Surgery (Department of Dentistry of the University of Cincinnati), were held at the Odeon, Cincinnati, Ohio, on Tuesday evening, April 14, 1896.

The annual address was delivered by Rev. E. Trumbull Lee, D.D., and the class address by Fenimore Roudebush, D.D.S.

The number of matriculates for the session was two hundred and fifteen.

The degree of D.D.S. was conferred on the following graduates by Dr. Frank A. Hunter, President of the Board of Trustees:

| NAME. | RESIDENCE. | NAME. | RESIDENCE. |
|---|---|---|---|
| Virgil V Adkins | West Virginia | Alonzo M Miller | Indiana |
| Wm A Blackwell | Illinois | John W Miller | Ohio |
| James E Boyd | Ohio | Thomas M Pearce | Kentucky |
| Leon E Boyd | Ohio | Thomas S Phillips | Ohio |
| James T Campbell | Kentucky | Sam Shaw Phister | Kentucky |
| Lewis C Cowden | Ohio | John S Recob | Ohio |
| Charles S Dunham | Colorado | Fred F Rice | Ohio |
| Charles V Dye | Ohio | Warren A Robison | Indiana |
| Reichart Erdman | Ohio | Fenimore Roudebush | Kentucky |
| William B Forsythe | Ohio | Lewis L Ross | Kentucky |
| Wilson Foster | Ohio | Sallie K Runyon | Indiana |
| Frank W Frey | Ohio | Oscar F Schleef | Missouri |
| John C Goreis | Ohio | Herbert A Schafer | Ohio |
| E E Hackleman | Indiana | Milton H Schaffer | Ohio |
| Frank Harding | Indiana | Edward C Sherman | Ohio |
| Wm F Heisel | Ohio | James H Shields | Indiana |
| Sam'l D Hockman | Ohio | Carl M Simonson | Minnesota |
| Harry S Hopkins | Indiana | William H Stacy | Kentucky |
| Clair M Hulley | Ohio | Florence E Taylor | Ohio |
| Burt L Lackey | Ohio | Albert E Thompson | Kentucky |
| Joseph Lang | Ohio | Arthur P Walton | Kentucky |
| Arthur J Markley | Kentucky | Leonard H Wilson | Canada |
| Karl L Mayers | Ohio | Theodore C Workman | Ohio |
| William B McKee | Ohio | | |

### Columbian University—Dental Department.

The ninth annual commencement exercises of the Dental Department of the Columbian University were held in Metzerott Hall, Washington, D. C., Tuesday, May 5, 1896, at 8 o'clock, P. M.

The address to the graduates was delivered by Professor L. C. F. Hugo, D.D.S., and the valedictory by Joseph L. Egan, D.D.S.

The degree of D.D.S. was conferred on the following graduates by B. L. Whitman, D.D., President of the University:

| NAME. | RESIDENCE. | NAME. | RESIDENCE. |
|---|---|---|---|
| R V Barry | Dist. of Columbia | L J Broughton | North Carolina |
| E F Concklin | Rhode Island | Joseph L Egan | Connecticut |
| W S Hall | Alabama | Harry A Jelly | Maryland |
| L Jordan, M.D. | Mississippi | Hubert L King. | Dist. of Columbia |
| S C Luckett | Texas | John A Moore | Indiana |
| H B Moore | Dist. of Columbia | J R Stewart | Virginia |
| R E L Wiltberger | Dist. of Columbia | J L Whiteside | Maryland |

### Atlanta Dental College.

The annual commencement exercises of the Atlanta Dental College were held at the Grand Opera House, Atlanta, Ga., on Thursday evening, March 26, 1896.

The valedictory was delivered by C. E. Hines, and the address to the graduating class by Dr. V. E. Turner.

The number of matriculates for the session was two hundred and two.

The degree of D.D.S. was conferred on the following graduates by Dr. J. S. Hopkins, Vice-President of the Board of Trustees:

| NAME. | RESIDENCE. | NAME. | RESIDENCE. |
|---|---|---|---|
| E T Boothe | Georgia | R J Kennedy | Georgia |
| C W Ball | Georgia | J W Kelly | South Carolinia |
| R W Bragg | Virginia | Edgar Jackson | Georgia |
| C W Baker | Alabama | C C Jordan | Georgia |
| L W Burt | Georgia | W A McGee | Georgia |
| E C Colley | Louisiana | J M McGee | Georgia |
| Peter Colcough | Georgia | W M McRae | Georgia |
| P M Caillouet | Louisiana | W K Meeks | Georgia |
| H J Cunyus | Texas | R M Mason | Georgia |
| C V DeLoach | Georgia | J W Manning | Alabama |
| W R Davis | North Carolina | W B Nichols | Mississippi |
| E P Frazier | South Carolina | J D Adeneal | Mississippi |
| A G Gilder | Texas | W G Sharp | Georgia |
| E F Gillon | Massachusetts | D A Spence | Georgia |
| H C Hopkins | Georgia | J G Reid | Georgia |
| C T Hall | Georgia | H M Todd | South Carolina |
| C A Holyendorf | Georgia | G E West | Mississippi |
| W P Hansard | Georgia | L J White | South Carolina |
| C E Hines | Mississippi | J T Wester | Georgia |
| J A Hightower | Alabama | D M Yates | Alabama |

### Cincinnati College of Dental Surgery.

The annual commencement exercises of the Cincinnati College of Dental Surgery were held at Sinton Hall, Cincinnati, O., on Thursday evening, April 2, 1896.

Remarks were made by the Dean, Professor G. S. Junkerman, M.D., D.D.S., and the valedictory address was delivered by Professor William O. Sproull.

The number of matriculates for the session was thirty-eight.

The degree of D.D.S. was conferred on the following graduates by Hon. Francis B. James, LL.B., President of the Board of Trustees:

| NAME. | RESIDENCE. | NAME. | RESIDENCE. |
|---|---|---|---|
| Jasper N Bradford | Kentucky | Theodore C Gelhaar | Wisconsin |
| William K Chambers | Indiana | Beaumont H Kaighn | Kentucky |
| Ollie D Doran | Iowa | Frank R Smith | Ohio |
| Aaron Grodsky | Ohio | Leslie D Spence | Ohio |

### New York College of Dentistry.

The thirtieth annual commencement exercises of the New York College of Dentistry were held in Chickering Hall, New York City, on Thursday evening, May 14, 1896.

The address to the graduates was delivered by Professor J.W. Dowling, M. D., and the valedictory by Clark A. Heydon, jr., D.D.S.

The number of matriculates for the session was three hundred and thirty-seven.

The degree of D.D.S. was conferred on the following graduates by F. F. Vander Veer, President of the Board of Trustees:

L H Abel
C M Aden
C H Allen
C E Andelfinger
T R Augsburg
W S Barber
J B Besant
J T Brickell
B A Burns
R B Carr
H O Carson
A L Del Castillo
G R Clark
W S Currie
Washington Dailey
A M Desnoes
L R Devine
E A Du Brul
F S Downs
T S Dunning
J B Farrell
J H Finney
C E Fraser, jr
Wilson Fitz Randolph
C J Gies
F H Giesselmann
H F Hammer
E W Harlan
C F Harreus
Otto Herbhold
C A Heydon, jr
H K Howard
W A Kregeloh
M C Kohler
B G De Laval
F B Le Roy
R C M Lieneau
Adolph Lifshutz
Leo Mandelstamm
R B L Magruder
G W Marshall
Hector DeMarchena
F H Miller
A J McCarthy
J M McCormack
G H Muth
J H Osiatinsky
J G Payne
Sidney Phillips
H J Pillion
W A Sanderson
Moritz Schlesinger
H C Scobey
H M Shaley
DeWitt C Smith
Harris Smith
W M Smith
F B Spooner
G R Stein
W M Sullivan
R W Sweetser
Burton Talmage
J F Thompson
F L Tooley
Charles Vetter, jr
Emil Vejvoda
G W Wakeley
W S Waterbury
R S Watson
Armin Wald
W R Wengorovius, jr
I N Wheeler
W M Whitlock
F D Wygant

### Harvard Dental College.

The annual commencement exercises of the Harvard Dental College took place, in connection with those of the other Departments of Harvard University, at Boston, Mass., on Wednesday, June 24, 1896.

The degree of Doctor of Dental Medicine was conferred on the following graduates by the President, Charles W. Elliot, LL.D.:

| NAME. | RESIDENCE. |
|---|---|
| Francis H Barnard | Minnesota |
| Edgar C Bienemann | England |
| Asher H C Chase, Scs | Massachusetts |
| Ernest Howard Chute | Massachusetts |
| Charles Winfield Crane | Massachusetts |
| Harold DeWitt Cross | New Hampshire |
| John G Emery | Massachusetts |
| Edwin L Farrington | Massachusetts |
| Adelbert Fernald | New Hampshire |
| Gwido Webster Gilbert | Massachusetts |
| Henry Sargent Gilman | Massachusetts |
| Henry West Haley | Massachusetts |
| Harvey W Hardy | Massachusetts |
| Robert John McMeekin | Massachusetts |
| Henry Horrill Haynes | Maine |
| Jacob Francis Martin | Massachusetts |
| Edward W Matthews | Massachusetts |
| Chas Everett Monroe | Massachusetts |
| Thomas Kennedy Ross | Massachusetts |
| P J A Stadelmann | Germany |
| G Irving Sweet | Rhode Island |
| Chas Frederick York | Massachusetts |

### University of Tennessee—Dental Department.

The annual commencement of this Department was held in the Vendome Building, March 27, 1896. Prayer was offered by Rev. Dr. Morris, of the Methodist Church, after which Dr. W. O. Menzies, of Tennessee, was introduced, and delivered the class valedictory, which was a gem in its way, and elicited the admiration of all present.

Professor Jordon of the Academic Department was introduced, and addressed the class in brief but well-chosen words, after which, in the absence of Dr. Dabney, the President of the University—who was unavoidably absent or detained—he conferred the degree of D.D.S. on the following persons:

Dr. L. G. Nowell delivered the charge to the class.

There were in attendance, in the school during the term, fifty-four students.

| NAME. | RESIDENCE. |
|---|---|
| J F Coyle | Alabama |
| E B Fuller | Texas |
| James Harmon | South Carolina |
| A F Hudson | South America |
| R Lee King | Mississippi |
| Z W Moss | Missouri |
| W P Menzies | Tennessee |
| S P Myers | Pennsylvania |
| J L Pennington | Tennessee |
| H L Sanders | Tennessee |
| F E Sandusky | Tennessee |

### University of California—College of Dentistry.

The annual commencement of this institution was held at Odd Fellows' Hall, San Francisco, Cal., on Thursday evening, June 18, 1896, at 8 o'clock.

The address on behalf of the faculty was delivered by Professor J. N. Williamson, M. D.

Hon. Martin Kellogg, A.M, LL.D., President of the University, conferred the degree of D.D.S. on the following named persons:

| NAME. | RESIDENCE. | NAME. | RESIDENCE. |
|---|---|---|---|
| William H Abbay | California | George Morgan Harris | California |
| Henry Abrahm | California | Alex Hamilton Hawley | California |
| George Abrams | California | George Henry Haynes | Brit. Columbia |
| Marilda Jane Ayers | California | Samuel William Hilliard | California |
| Frederick Guernsey Baird | California | Guy Brown Husted | California |
| Mary Louise Baird | California | Konrad Magnus Lundborg | California |
| Bertram Carl Boeseke | California | Stephen Chol Maynard | California |
| Franklin Calvin Bonnel | California | Naoma Gae MacDonald | California |
| Charles Harold Bowman | California | Robert B McNutt | California |
| Albert Caferata | California | Thomas Snelling Morden | Idaho |
| Paul Tulane Carrington | California | Fred Gustave Pless | California |
| Harry George Chappell | California | Charles Bruce Porter, jr | California |
| William Nathan Clark | California | Joseph Ignatius Richards | California |
| Frank Herman Cranz | California | Leon Joseph Roth | California |
| Jacob Cohn | Idaho | Miss Anna Martin Sawyer | California |
| Stephen J Cunningham | California | John Herbert Seager, A.B | Turkey |
| William Edward Davis | California | Frank Joseph Smith | California |
| James Morton Forrest, jr | California | George Edward Stallman | California |
| Arthur A Fowler | California | Benjamin Mitchell Stich | California |
| H Edward Gedge | California | Montgomery Thomas | California |
| Amy Maxted Gilbert | New South Wales | Oscar Tobriner | California |
| Charles Daniels Gilman | California | Clifford Todd | California |
| Lawrence Greenbaum | California | William Henry Ware | California |
| Richard George C Harms | California | Lauren David Webster | California |
| Charles Edwin Hart | California | Clyde Allen Weldon | California |
| Arthur Philip Harth | Oregon | Edward Wm Westphal | California |

### University of Michigan—College of Dental Surgery.

The annual commencement of the University of Michigan was held in the hall of the University June 25, 1896.

The annual address was delivered by Charles Candel Adams, LL.D., President of the University of Wisconsin.

Upon this occasion were the commencement exercises of all Departments of the University.

There were, in the Dental Department, during the year just closed, one hundred and eighty-nine students—candidates for graduation, 58.

The degree of D.D.S. was conferred by Dr. James B. Angel, President of the University, upon the following named persons:

| NAME. | RESIDENCE. |
|---|---|
| Elmer Harry Argetsinger | Minnesota |
| Frank Charles Arnold | Michigan |
| Jay Cyrus Arnold | Michigan |
| Frank Miller Bacon | Michigan |
| Clarence Harvey Bailey | Michigan |
| John Wesley Bass | Indiana |
| Eddie W Brown | Michigan |
| Edward Dancey Brown | Ontario |
| Robert R Buckthorpe | Illinois |
| Harry Sizer Buell | Michigan |
| George Franklin Burke | Michigan |
| Willis Hezekiah Buttolph | Michigan |
| Jessie Estelle Castle | Michigan |
| James Nelson Clarke | Michigan |
| Charles William Cleaver | Michigan |
| Jonathan P Collett, B.S. | N. Nor. Univ |
| Irving William Copeland | Michigan |
| Ernest Frank Day | England |
| Edwin Victor Deans | New York |
| Charles Alphonso Devlin | California |
| Stanford James Farnum | Michigan |
| Stanley Ammon Farnum | Michigan |
| Charles Frederick Fitch | New York |
| Fred Ansom Graham | Michigan |
| Fred Joseph Hale | Michigan |
| Hector Hillman | Michigan |
| Cleveland A Houghton | New York |
| Burton Truman Hunt | New York |
| Charles Lee Kemery | Michigan |
| Vernor Jay Lathrop | Michigan |
| John Adolph Lentz, LL.B. | Michigan |
| Howard Jos Livingston | Colorado |
| James White Lyons | Michigan |
| Thomas Stephen Mann | Michigan |
| Saml Stephen Mummery | Michigan |
| James Henry O'Toole | Michigan |
| Chas Augustus Phillips | Indiana |
| Ross Porter | Pennsylvania |
| Frank Glenn Powers | Michigan |
| Herman Prinz | Ohio |
| Charles A Ouackenbush | Michigan |
| James Robins | Ontario |
| William Howard Roper | Wisconsin |
| Thos Francis Sheridan | Michigan |
| Charles L Sherwood | Pennsylvania |
| Charles Eyster Slagle | Illinois |
| Albert Lyman Smith | Michigan |
| William Joseph Stapish | Michigan |
| Morley Punch. Templar | Ontario |
| Wilbur Townsend | Iowa |
| Albertus Van Ark | Michigan |
| Charles Alfred Wehe | Kansas |
| Ralph Levant Williams | New York |
| Raymond L Williams | Wisconsin |
| Robert Millard Woodin | Michigan |
| George Herbert Wooton | Michigan |
| John Alexander Wooton | Michigan |
| Percy Bennett Wright | Michigan |

### University College of Medicine—Dental Department.

The annual commencement exercises of the Dental Depart-partment of the University College of Medicine were held in the Academy of Music, Richmond, Va., on Wednesday, April 29, 1896.

The annual oration was delivered by ex-Governor Fitzhugh Lee, of Virginia.

The number of matriculates for the session was thirty-six.

The degree of D.D.S. was conferred on the following graduates by Stuart McGuire, M.D., Professor of Principles of Surgery:

The number of matriculates for the session was three hundred and twenty-three.

| NAME. | RESIDENCE. |
|---|---|
| F V Clarke | Virginia |
| W J Cowardin | Virginia |
| Geo D Farrow | Virginia |
| Frank Ferguson | South Carolina |
| M T Gay | Virginia |
| John H Hartman, jr | Virginia |
| W R Lyman | Virginia |
| J T Marshall | Virginia |
| E C McSparran | Virginia |
| Chas A Newland | Virginia |
| A C Oppenheimer | Virginia |
| Wm Pilcher | Virginia |
| E U Potter, jr | Virginia |

### University of Denver—Dental Department.

The annual commencement of the Dental Department of the University of Denver was held Thursday evening, April 16, 1896.

Total number of matriculates twenty-eight.

An address to the graduates in Dentistry was delivered by Professor J. M. Norman.

Also an address of a more general character was delivered by Rev. James H. Ecob, D.D.

Chancellor McDowell conferred the degree of D.D.S. on the following candidates:

| NAME. | RESIDENCE. | NAME. | RESIDENCE. |
|---|---|---|---|
| Louis Philip Paul | Colorado | Charles Lincoln Mercer | Colorado |
| Sallie Sherman Hanley | Unknown | Frank Miller Peck | Colorado |
| Alice Gertrude Grant | Massachusetts | Elliott Sangster Miller | Colorado |
| Irvine Hunter | Colorado | Charles Eugene Baur | Colorado |

### Boston Dental College.

The annual commencement of the Boston Dental College was held in the Berkley Temple, June 19, 1896.

The general address was delivered by E. N. Capypin, D.D., President of Tuft's College.

The valedictory was delivered by John P. Brigham, D.D.S.,

The degree of D.D.S. was conferred on the candidates by I. J. Wetherby, D.D.S., President of the College.

There were enrolled in the college during the term just closed, one hundred and eighty-nine students.

The following are the names of the graduates:

| NAME. | RESIDENCE. | NAME. | RESIDENCE. |
|---|---|---|---|
| Mary E Alleyne | Prince Edward's I. | George Otis Irish | Massachusetts |
| Joseph L Bellefleur | Massachusetts | Charles Timothy Kinsman | Vermont |
| John Morton Bellews | Massachusetts | Wm Henry Langdon | Massachusetts |
| John Perkins Brigham | Vermont | Jno Roderick McKinnon | Nova Scotia |
| Edward James Cowell | Massachusetts | Daniel L Manchester | Massachusetts |
| George William Day | Massachusetts | Chas Ebenezer M'Intire | Massachusetts |
| James William Devlin | Massachusetts | George Martin Mayers | Massachusetts |
| Albert James Duffy | New Hampshire | Edwd George Marshman | Connecticut |
| John Hancock Eaton | Massachusetts | Franklin Leroy Mears | Massachusetts |
| Dana Jos Edmonds | New Hampshire | Stanley Colebrook Neals | Nova Scotia |
| Chas Frederick Erickson | Connecticut | Etta May Ober | New Hampshire |
| Edmond Francis Flynn | Massachusetts | Samuel Ed O'Donnell | Massachusetts |
| Joseph Michael Gaffey | Massachusetts | Danzy Russell Povey | Massachusetts |
| Burleigh Childs Gilbert | Massachusetts | John Francis Riley | Massachusetts |
| Frank Leslie Gower | Massachusetts | Frank Byron Sharpe | New Brunswick |
| Arthur Wardwell Green | Massachusetts | George Wakefield Soule | Maine |
| Wm Henry Harrington | Rhode Island | Walter Eugen Tuttle | Maine |
| Winfield E Hanson | Massachusetts | Jno Andrew L von Betzen | Nova Scotia |
| Francis Patrick Harris | Massachusetts | | |

### Louisville College of Dentistry.

The annual commencement exercises of the Louisville College of Dentistry was held at Macauley's Theater on the —— in connection with the commencement of the Hospital College of Medicine. The exercises of the Dental College were held first.

Prayer was offered by Rev. J. Earnest Thacker.

Professor P. Richard Taylor, M. D., presented the following named persons for the degree of D.D.S.:

Professor Peabody, after a very happy address, conferred the degrees on the candidates.

Prizes were awarded as follows:

S. Marlowe, first honor medal; Thomas G. Vanhook, second honor medal; W. A. Fisher, for proficiency in Operative Surgery. Wm. G. Scott was also awarded a medal.

The valedictory address was delivered by Dr. M. J. Dryer.

Wm H Bartholomew, jr
Arthur Burton
Steve O Carter
Joseph Childers
Rutherford D Coffman
Evans Dazey
Charles W Dean
Melvin J Dryer
Eugene S Espy
Wilbur A Fisher
Oliver E Hawn
Galen Owen Loomis
Charles Servoss Jackson
Oscar Jones
Gilbert O Lee
Searcy Marlowe
Harold McCrory
Frank A Meder
Eugene Motley, M.D.
G August Pielemier
Philip J Pollock
Theodore F Roemele
Wm Francis Ross
John Elmore Ruby
Geo P Sadtler
Wm G Scott
Benjamin S Shinness
Joe C Shumaker
Albert H Speer
Thos G Vanhook
Price Wayne Wells
Allie B Wilburn.

### National University—Dental Department.

The twelfth annual commencement exercises of the Dental Department of the National University were held, in connection with those of the Medical Department, in Metzerott Hall, Washington, D. C., on Thursday evening, June 11, 1896.

The address to the graduating classes was delivered by Professor W. D. Bigelow, Ph.D., and the valedictory address by Edgar W. Watkins, M.D.

The number of matriculates for the session was forty.

The degree of D.D.S. was conferred on the following graduates of the dental class:

| NAME. | RESIDENCE. |
|---|---|
| Peter T Kirwan | New York |
| Edmund O Pigeon | New York |
| Joseph Pospisiel, M.D | Wisconsin |
| J Vernon Priddy | Dist. of Columbia |
| E E Rankin | Indiana |
| A B Stine | Pennsylvania |

### University of Pennsylvania—Department of Dentistry.

At a public commencement of the University of Pennsylvania, held Thursday, June 11, 1896, at the American Academy of Music, Philadelphia, Pa., the degree of D.D.S. was conferred by Charles C. Harrison, A.M.. LL.D., Provost, on the following candidates:

| NAME. | RESIDENCE. | NAME. | RESIDENCE. |
|---|---|---|---|
| Francis S Avil | Pennsylvania | Wm E Kiner | Pennsylvania |
| Fayette H Beard | Pennsylvania | Frederick F Knapp | Pennsylvania |
| Chas D Bender | Pennsylvania | Allison R Lawshe | New Jersey |
| John C Benz | New York | Walter W McCarty | Iowa |
| Charles S Blaker | Pennsylvania | Wm C Marsh | Massachusetts |
| Carl Bonten | Germany | Sherman M Matheny | Pennsylvania |
| Alfred E Bull | Pennsylvania | Lucius P Meaker | New York |
| Guy M Byrkit | Iowa | Ludwig Moestue | Norway |
| Ernest E Cardwell | England | Edward H Moore | Pennyslvania |
| J Edward Chace | Florida | Daniel K Musser | Pennsylvania |
| C A Christine, jr | Pennsylvania | John H O'Hagan | New York |
| Samuel T Clay | Pennsylvania | J Howard Ozias | Pennsylvania |
| Thomas J Clemens | Pennsylvania | George C Parry | New York |
| Charles W Colborn | Pennsylvania | C Gilbert Pearse | England |
| Albert L Connelly | Pennsylvania | Fred E Pottle | Maine |
| J Harold Cornell | Pennsylvania | Harry S Pritchett | Minnesota |
| Nile Crist | Pennsylvania | Charles W Rabb | Pennsylvania |
| Clyde Decker | New Jersey | Edward D Rank | Pennsylvania |
| Herbert B Denis | New Jersey | Harry F Reynolds | Pennsylvania |
| G Henry S Dobbyn | Australia | Charles Ruegg | Switerland |
| James A Dowden | New York | Morris I Schamberg | Pennsylvania |
| John Earley, jr | Pennsylvania | Frank H Schultz | Ohio |
| Wm S Eisenhart | Pennsylvania | Joseph Schuger | Hungary |
| Alfred B Ellen | Pennsylvania | Blake A Sears | New York |
| Morris F Elliott | Pennsylvania | James C Seymour | Nebraska |
| Malcolm Ellis | Brazil | Herbert H Shapard | Texas |
| William Farrar | Ohio | Vane G Shiffler | Pennsylvania |
| Carl Feibusch | Germany | George C Speirs | Connecticut |
| T Stanley Gamble | Pennsylvania | Nathan P Stauffer | Pennsylvania |
| V Walter Gilbert | Pennsylvania | P Albert Taeuber | Switzerland |
| Nicholas J Graeber | Pennsylvania | William D Tracy | Massachusetts |
| Wm C Graham | Ohio | Otto Venn | Germany |
| Pearl Z Grey | Ohio | S Albert Walker | Pennsylvania |
| G Clifton Guest | Pennsylvania | Elbert J Weaver | Wisconsin |
| John P Headridge | England | W L Webster | New York |
| Harry B Hickman | Delaware | Charles M Wharton | Delaware |
| J Ernest Keeler | Pennsylvania | Wm Yeakel, A.B | Pennsylvania |

### Western Reserve University—Dental Department.

The annual commencement of the Dental Department of Western Reserve University was held in Association Hall, Cleveland, Ohio, May 19, 1896, at 7:30 P. M.

The principal address was given by Dr. J. M. Buckley, D.D., of New York City.

The number of matriculates for the session was fifty-three.

The degrees were conferred by the President, Charles F. Thwing, D.D., upon the graduates, as follows: Wm. G. Ebersole, Frank H. Fagan, Joseph W. George, William O. Haldy, Charles E. Hurd, John W. L. Thomas, John S. Van Meter, all of Ohio.

